# The start of sexual health curriculum development and evaluation at Stellenbosch University

**DOI:** 10.4102/phcfm.v15i1.3825

**Published:** 2023-03-08

**Authors:** Heidi van Deventer, Michael W. Ross, Jantien Thomson, Marlena du Toit, Mieke Poelsma, Marie Pienaar, Andre van der Merwe, Matthys H. Botha

**Affiliations:** 1 Division of Urology, Faculty of Medicine and Health Sciences, Stellenbosch University, Cape Town, South Africa; 2 Department of Family Medicine and Community Health, Institute of Sexual and Gender Health, University of Minnesota, Minneapolis, MN, United States; 3 Private Sexology Practice, the Netherlands; 4 Department of Obstetrics and Gynaecology, Faculty of Medicine and Health Sciences, Stellenbosch University, Cape Town, South Africa

**Keywords:** curriculum development, sexual health course, medical curriculum, taking a sexual history, sexual health, SHEPS

## Abstract

**Background:**

Stellenbosch University’s (SU) Faculty of Medicine and Health Sciences (FMHS), developed a sexual health course to be integrated throughout the revised medical curriculum.

**Aim:**

To use the Sexual Health Education for Professionals Scale (SHEPS) to gather baseline and future follow-up data to inform curriculum development and evaluation.

**Setting:**

The first-year medical students (*N* = 289) of the FMHS SU.

**Methods:**

The SHEPS was answered before the start of the sexual health course. The knowledge, communication and attitude sections were answered with a Likert-type scale. Students had to describe their perceived confidence in their knowledge and communication skills to care for patients within specific sexuality-related clinical scenarios. The attitude section measured the students’ level of agreement or disagreement on sexuality-related opinion statements.

**Results:**

The response rate was 97%. Most students were female, and 55% of the class were first taught about sexuality in the age group 13–18 years. The students had more confidence in their communication skills than knowledge before any tertiary training. The attitude section revealed a binomial distribution, ranging from acceptance to a more restrictive attitude towards sexual behaviour.

**Conclusion:**

It is the first time the SHEPS has been used in a South African context. The results provide novel information about the range of perceived sexual health knowledge, skills and attitudes of first-year medical students before they start tertiary training.

**Contribution:**

Findings from this study will guide content development and evaluation of the sexual health course at the institution where the study was conducted, as well as allow for culture sensitive education.

## Introduction

The late Archbishop Desmond Tutu referred to South Africa (SA) as the rainbow nation – a country with 11 official languages and a multitude of different cultures. The influence of religion and culture on sexual and reproductive behaviour and healthcare utilisation is supported by research.^1,2^

The current comprehensive sexual health education (CSE) program in schools, developed by the Department of Basic Education (DBE), strongly advocates abstinence among young people^3^ regardless of the cultural backgrounds or experience of each learner. This approach is shown to be ineffective and even harmful to young people who are already sexually active, who are gay, lesbian, bisexual, transgender, queer, intersex, and asexual (LGBTQIA+) or who have experienced sexual abuse.^4,5,6,7,8^ However, the DBE is at the same time committed towards contributing to the prevention and management of human immunodeficiency virus (HIV), sexually transmitted infections (STIs) and tuberculosis (TB) by ensuring that learners, educators, officials and parents are informed and equipped to decrease risky sexual behaviour and gender-based violence (GBV) among young people.^3^

In the Western Cape Health Vision 2030, it is stated that ‘to become a healthy nation, South Africans need to make informed decisions about what they eat, whether or not they consume alcohol, and their sexual behaviour, among other factors’. Primary health care providers who have knowledge and skills in offering sexual health care can help their patients and communities to make well-informed decisions on sexuality-related issues.

Medical curricula in the Western world are known to limit their focus to reproductive health and STIs.^9,10,11^ However, the Word Health Organization (WHO) suggests that sexuality and sexual relations are central to both reproductive and sexual health, but most sexual activity is not directly associated with reproduction and is of relevance throughout a person’s lifespan, so sexual health may be considered to be a broader concept.^12^

Undergraduate sexual health education, such as sexuality and sexual behaviour, including the anatomy, physiology and psychosocial aspects of sexual function and dysfunction, does not form part of the syllabus during the 6 years of medical training.^13^ Recently, the University’s Faculty of Medicine and Health Sciences (FMHS) revised its existing medical curriculum, which created an opportunity to develop a course on sexual health education for medical professionals to be included in the revised medical curriculum.

The previous curriculum of the FMHS focused on teaching basic applied medical sciences, emphasising mainstream disciplines and was tailored to diagnosing and managing disease in a resource-poor country. A gynaecologist offered two lectures in the 2nd and 5th year, and there were some tutorials by urologists during the clinical rotation of students. Sexual history taking was not practiced, and no case studies were offered. The lack of training in sexual history taking of a patient contributes to it not being a routine consideration of doctors at the primary care level to ask about their patient’s sexual health.^14,15^

About 80% of the SA population depends on the public sector primary health care service.^16^ Because of the importance of a well-functioning primary health care system, medical students start rotating through primary health care clinics and work in primary health care teams as soon as their first-year of medical school and continue throughout the 6 years of medical training.^17^

Pretorius et al. observed that, when a doctor is uncomfortable talking about sex, it can prevent patients from sharing concerns about their sexual health. In their research, they also reported that patients experience judgement about their sexual behaviour by their doctors. The lack of training during medical school can lead to personal biases that influence the professional attitude of the doctor. These biases could stem from differences in culture, religion, societal conditioning, experience and other factors.^14,15^

In response to her findings, Pretorius also recommends that ‘the ideal would be if primary care doctors and family physicians are trained to initiate sexual history taking for sexual dysfunction’.^14,15^

The primary purpose of the newly developed sexual health course is to enable students to become more comfortable with taking an appropriate sexual history from their patients.^18^ Secondary outcomes are to encourage self-awareness of the students, becoming cognisant of factors creating possible inherent bias related to sexual health care of their patients.^18,19^

The sexual health course development team plans to integrate the course throughout the 6 years of medical training by teaching specific aspects of sexual healthcare as the students’ progress through the curriculum, gradually building on the comfort, skills and knowledge gained in the previous years.

The new sexual health course was introduced in February 2022 as part of curriculum renewal of the FMHS. To guide the course development, a validated, contemporary questionnaire developed by Bayer and Shindel,^20^ was used. The questionnaire has been used in various countries, including the USA and Tanzania,^20^ and is known as the Sexual Health Education for Professionals Scale (SHEPS).

This paper aims to describe the baseline results of the first SHEPS and the demographics of the current first-year medical students, as part of a broader study including follow-up measurements of the SHEPS, thereby providing a starting point for curriculum development at our institution. This offers the opportunity for culture sensitive education taking into account existing levels of knowledge and comfort of students on specific sexuality-related issues.

## Research methods and design

### Study setting

The population constitutes students in their first-year of a six-year medical degree (*N* = 289) of the FMHS, Stellenbosch University, Tygerberg Medical Campus, Western Cape, South Africa.

### Study design and overview of data collection

The primary study design is a longitudinal analytical study, using repeated measures of the same survey (SHEPS) on the same group of students for the analysis. The first (baseline) survey was completed online and in English in February 2022 and forms part of the mandatory component of the course. The first-year students completed the SHEPS as the first task of the sexual health course.

The questionnaire was included because of its dual purpose: to serve as an educational development tool and to be used for research. If students did not consent to have their data processed in the research project, completing the SHEPS was still compulsory as part of the sexual health course and their individual development. An informed-consent document was included in the accompanying email before the start of the questionnaire, providing an option for the student to consent for their data to be used for research purposes or to opt out.

A sponsorship of 100 books containing appropriate content for first-year medical students incentivised students to participate in the research. The 100 books were offered in a random draw.

The questionnaire evaluates students’ perceived level of comfort with specific sexuality-related issues that could be encountered during the taking of a sexual history. It consists of three components: skills, knowledge, attitude and an extra part that assesses demographic differences. The skills component assesses the student’s perceived ability to communicate, assess and discuss sexuality and sexuality-related topics (in different scenarios). The knowledge component assesses the perceived knowledge to care for patients in these scenarios.^21^ The attitude component assesses students’ attitudes regarding statements about sexuality and sexuality-related topics. The last element of the questionnaire collects demographic and personal information adapted to the context of the medical school.

### Statistical analyses

This article reports the first completion of the SHEPS in a cross-sectional, descriptive format.

The demographic information of the students will be represented in descriptive analyses and include age, gender, year of study, sexual orientation, relationship status, exposure to any sexual health education before medical school and where and at what age the sexual health education occurred (Table 1).

**TABLE 1:** Demographics of first-year medical students in 2022 (*N* = 281).

Demographics	*n*	%
**Current sex or gender identity? (*n* = 281)**		
Male	65	23
Female	211	75
Transmale or Transman	0	0
Transfemale or Transwoman	0	0
Genderqueer	2	1
Prefer not to answer	2	1
Other	1	0
**Which best describes you?**		
Asexual	6	2
Bisexual	14	5
Gay	8	3
Lesbian	3	1
Heterosexual or Straight	235	84
Unsure	10	4
Other	1	0
Prefer not to answer	4	1
**What is your relationship status?**		
Single	203	71
Dating casually	17	6
In a relationship but not married	54	19
Married	4	1
Divorced or Separated	0	0
Widowed	0	0
Other	0	0
Prefer not to answer	7	2
**How old were you when you were first taught about sexuality?**		
0–5 years	1	1
6–12 years	99	35
13–18 years	154	55
18+ years	6	2
Don’t know	18	6
Prefer not to answer	3	1

The questions of the three sections of the questionnaire (communication skills, knowledge and attitude) are all answered on a 7-point Likert-type scale, according to the format of the standardised SHEPS. The original SHEPS was used, with only a few adaptations to the demographic section, but no changes were made to the communication, knowledge and attitude sections. Results have been described by the mean and standard deviation for each question. Pearson’s correlation coefficient was used to measure the level of linear correlation between knowledge and communication, thereby testing for a significant relationship between the two variables. Communication skills and knowledge results are presented in Table 2, and the attitudes section has been described in Table 3.

**TABLE 2:** Summary of knowledge and communication results (*N* = 281).

Question	Mean†	s.d.†	*n*	Mean‡	s.d.‡	*n*	Paired two-tailed *t*-test (*p*)	Pearson’s correlation (*r*)§	*n*
1. … the parents of a fetus or newborn with a disorder of sex development (e.g., ambiguous genitalia)	5.04	2.05	246	2.80	1.76	269	0.0000	0.43	236
2. … a pre-pubescent child (i.e., masturbation, genital exploration of self and other children, questions about sex, ‘birds and the bees’) (UN, 2013)	3.27	1.91	275	2.86	1.71	272	0.0033	0.49	268
3. … a pubescent person (i.e., body changes with puberty, becoming sexually active, decision making)	2.60	1.63	278	2.14	1.35	279	0.0000	0.44	277
4. … a young (18–40 years) adult (i.e., promoting sexual wellness)	3.05	1.85	276	2.14	1.37	279	0.0000	0.41	274
5. … a middle-aged (41–65 years) adult (i.e., promoting sexual wellness)	3.99	1.94	269	3.08	1.73	269	0.0000	0.52	261
6. … an older (>65 years) adult (i.e., changes in sexuality with ageing)	4.59	1.92	258	3.47	1.89	269	0.0000	0.51	252
7. … a person with a mental disability (e.g., Downs syndrome, schizophrenia, traumatic brain injury	4.90	1.89	252	3.05	1.77	262	0.0000	0.47	238
8. … a person with a physical disability (e.g., cerebral palsy, spinal cord injury, amputations)	4.68	2.02	256	2.69	1.69	274	0.0000	0.42	251
9. … a person with sexual problems/dysfunctions or concerns	4.82	1.95	260	2.89	1.64	274	0.0000	0.47	255
10. … a person with sexual problem(s) related to a medical, pharmacological, or surgical treatment	4.90	2.10	248	2.66	1.69	273	0.0000	0.40	244
11. … a person whose gender and/or sex is different from your own	3.05	1.85	276	2.22	1.47	279	0.0000	0.50	275
12. … a person whose gender is the same as your own	2.24	1.67	279	1.52	1.03	280	0.0000	0.46	278
13. … a person who is transgender or genderqueer	4.14	2.08	260	2.74	1.89	260	0.0000	0.57	251
14. … a person who identifies as heterosexual	2.58	1.85	275	1.84	1.38	278	0.0000	0.62	274
15. … a person who identifies as non-heterosexual (e.g., lesbian, gay, bisexual, something else)	3.49	2.06	263	2.35	1.63	266	0.0000	0.57	256
16. … a person who identifies as asexual	3.80	2.11	250	2.49	1.74	260	0.0000	0.57	242
17. … a person who engages in non-normative sexual practices (e.g., sadomasochism, paraphilia or fetish)	5.05	1.90	231	3.77	1.97	242	0.0000	0.59	214
18. … a person who masturbates	3.36	1.95	264	2.50	1.62	262	0.0000	0.62	253
19. … a person who engages in sex with a committed partner (i.e., dyadic relationship)	2.80	1.82	272	1.79	1.30	277	0.0000	0.48	270
20. … a person who engages in casual sex (e.g., hookups, one-night stands)	3.34	1.84	268	2.30	1.51	272	0.0000	0.45	265
21. … a person who engages in transactional sex (e.g., sex work, prostitution, etc.)	4.07	1.91	257	2.95	1.74	259	0.0000	0.44	244
22. … a person who engages in sex with a person other than a partner in a dyadic relationship WITHOUT the other partner’s knowledge or consent (e.g., ‘cheating’)	4.24	1.99	250	3.48	1.88	259	0.0000	0.58	239
23. … a person who engages in sex with a person other than a partner in a dyadic relationship WITH the other partner’s knowledge and consent (e.g., ‘open relationship’)	3.89	1.90	255	2.79	1.76	262	0.0000	0.51	243
24. … a person who is coercive or abusive to their sexual partner(s)	4.80	1.89	238	4.29	2.05	226	0.1498	0.48	207
25. … a person who is coerced or abused by their sexual partner(s)	3.93	1.94	259	2.87	1.84	262	0.0000	0.49	249
26. … a person with questions about safer sex and sexually transmitted infections	2.93	1.85	277	1.65	1.23	281	0.0000	0.44	277
27. … a person infected with the HIV	3.47	2.02	270	1.97	1.39	272	0.0000	0.47	264
28. … a person who has a sexually transmitted infection OTHER than HIV	3.78	2.04	265	2.06	1.41	271	0.0000	0.43	260
29. … a person who desires contraception	2.73	1.74	273	1.61	1.13	280	0.0000	0.49	272
30. … a person who wishes to become pregnant or impregnate a partner	3.18	1.93	271	1.72	1.28	279	0.0000	0.42	269
31. … a person seeking an abortion	4.23	2.01	256	2.84	1.88	253	0.0000	0.46	239
32. … a person with conservative sociocultural beliefs about sexuality	3.69	1.89	266	2.52	1.56	269	0.0000	0.49	259
33. … a person with liberal sociocultural beliefs about sexuality	3.65	1.96	259	2.48	1.67	269	0.0000	0.52	253
34. … a person with religious/spiritual convictions about sexuality (in this context it refers to persons whose convictions stem from an organised religious group such as Catholicism, Islam, or Judaism)	3.71	2.01	266	2.42	1.62	273	0.0000	0.53	263
35. … a person who informs you of a topic that requires mandatory reporting (e.g., STI, threat of harm to others, etc.)	3.86	2.09	258	2.70	1.77	267	0.0000	0.46	250
36. … a person whose values pertaining to one or several aspects of sexuality are in conflict with your own	3.96	1.85	256	2.96	1.58	264	0.0000	0.51	244
37. … a person who requires referral for more specialised sexual healthcare	3.79	2.09	262	2.17	1.59	276	0.0000	0.48	258

Note: (1) Very confident; (2) Moderately confident; (3) Slightly confident; (4) Neither confident nor unconfident; (5) Slightly unconfident; (6) Moderately unconfident; (7) Very unconfident. The questions in this table are taken from Roth Bayer C, McKool M, Shindel A. Sexual Health Education forProfessionals Scale (SHEPS) in Ross et al. Evaluation of an assessmentinstrument for a sexual health curriculum for nurses and midwifery studentsin Tanzania: The Sexual Health Education for Professionals Scale (SHEPS).Appl Nurs Res. 2018;40:152–6. https://doi.org/10.1016/j.apnr.2018.01.005

HIV, human immunodeficiency virus; STI, sexually transmitted infections.

† , Do you feel confident that you have the knowledge to care for patients when discussing sexuality and sexuality-related topics with … ;

‡ , How confident are you in your ability to communicate, assess and discuss sexuality and sexuality-related topics with… ;

§ , Strength of positive correlation: 0.00–0.10 (negligible correlation), 0.10–0.39 (weak correlation), 0.40–0.69 (moderate correlation), 0.70–0.89 (strong correlation), 0.90–1.00 (very strong correlation).

**TABLE 3:** Summary of attitudes results (*N* = 281).

Statement	Mean	s.d.
C1. Educating teenagers on sex makes them more likely to do it	5.43	1.66
C2. Masturbation is a healthy part of human development	3.36	1.93
C3. Oral sex is an abnormal sexual practice	5.21	1.89
C4. Anal sex is an acceptable sexual practice	4.14	2.01
C5. It is okay to have sex before marriage	4.04	2.31
C6. Marriage should be only between a man and a woman	4.88	2.40
C7. I want to be a resource for my future patients with sexual problems	1.84	1.45
C8. It is okay to have a non-monogamous relationship if both partners agree to it	3.41	2.19
C9. It is not normal to be attracted to a person of the same sex	4.96	2.26
C10. I won’t be able to provide care for patients with sexual problems	6.47	1.09
C11. People who get sexual pleasure from inflicting and/or experiencing pain (sadomasochism) with consenting partners are sick	4.35	2.02
C12. Abortion should be available to women for whatever reason they choose	3.00	2.11
C13. Sex is not an issue that physicians should deal with in their practices	6.45	1.09
C14. Abortion is only allowable in special cases (e.g., rape, incest, threat to health of mother)	4.74	2.15
C15. Contraception should be easily available to anyone who wants it	1.47	1.19
C16. Sexual problems (e.g., erectile dysfunction, low sex drive, pain with sex) are serious issues that should be addressed)	1.58	1.06
C17. Being gay, lesbian, or bisexual is acceptable	2.45	1.94
C18. Healthy women always have a lower sexual drive than men	5.23	1.57
C19. People who contract sexually transmitted infections get what they deserve	6.52	1.04
C20. Abortion is murder	4.68	2.05
C21. People who are transgender deserve to receive care to help them conform to their chosen gender	2.47	1.86
C22. People should be allowed to marry someone of the same sex	2.50	1.96
C23. All pornography should be banned	3.42	2.06
C24. One can never be too old for sex	2.20	1.44
C25. I believe that I can use my human sexuality training effectively in a clinical setting	1.81	1.34
C26. I do not intend to use my human sexuality training in a clinical setting	5.95	1.65

Note: The results are based on the question: Please state your level of agreement or disagreement with the following statements. Please answer truthfully – there are no ‘wrong’ answers! The ratings are as follows: (1) Strongly agree; (2) Moderately agree; (3) Somewhat agree; (4) Neither agree nor disagree; (5) Somewhat disagree; (6) Moderately disagree; (7) Strongly disagree.

### Ethical considerations

Participating in the survey (for research purposes) was voluntary and anonymous. Although the SHEPS collected demographic and social data, it did not contain the students’ full names and birth dates, ensuring that the data collected was depersonalised. The students’ initials and month and year of birth were collected during the completion of the survey for matching future surveys and are kept in a password protected and firewalled file. The health research ethics committee of the university granted ethical approval: N21/08/073. The FMHS provided institutional permission.

## Results

We analysed the anonymous data of 281 first-year medical students who gave informed consent to use their anonymous data for research purposes. The total first-year medical students are 289, and thus the response rate was 97%.

The current results are the baseline descriptive and cross-sectional results, given that this is the first time the students will have answered the questions in SHEPS before the start of the sexual health course.

The mean (s.d.) age was 18.7 (1.9) years. Most first-year medical students indicated their gender identity as female (75%) and 23% as male. A further breakdown of gender identity and other demographic variables is included in Table 1.

### Summary of communication and knowledge results

The communication skills section of the SHEPS describes the confidence of the participants in their ability to communicate, assess and discuss sexuality and sexuality-related topics with their patients. The survey contains 37 scenarios involving a clinical content area, and the students had to respond according to a Likert-type scale ranging from 1 (very confident) to 7 (very unconfident), including 8 (don’t know) and 9 (prefer not to answer) which were treated as missing values. The knowledge section used the exact same list of 37 scenarios. Here, students had to describe their confidence level in their knowledge to care for patients when discussing sexuality and sexuality-related topics with specific clinical presentations. Table 2^22^ combines the communication and knowledge results, describing the mean and standard deviation of the responses to each answer. Correlations for individual items in knowledge and communications skills ranged from 0.41 (17% of variance in common) to 0.62 (38% of variance in common).

In the communication section, the students felt most uncomfortable when they had to communicate with people about a sexuality-related topic, who are in age groups other than their own, such as pre-pubescent children, middle-aged adults and especially older adults (>65 years), (questions 2, 5 and 6). They were also less confident in their ability to communicate about sexuality-related topics with a person with a mental disability, such as Down syndrome, schizophrenia or a traumatic brain injury (question 7). Other scenarios that the students were less confident about included a person who engages in non-normative sexual practices (question 17), a person who engages in transactional sex (question 21), a person who engages in sex with a person other than a partner in a dyadic relationship without the other partner’s knowledge or consent (question 22), a person who is coerced or abused by their sexual partner or partners (question 25), a person seeking an abortion (question 31) and a person whose values about one or several aspects of sexuality conflict with their own (question 36).

The question that the study participants were the most uncomfortable with was how to communicate about sexuality-related topics with a person who is coercive or abusive to their sexual partner or partners (question 24).

In the knowledge section, the topics that the students felt less confident about were similar to the communication section, with a moderately positive Pearson’s correlation coefficient. Specific topics elicited a low level of confidence in the student’s knowledge. These included questions about sharing knowledge with the parents of a foetus or newborn with a disorder of sex development (e.g. ambiguous genitalia) (question 1), a person with a physical disability (e.g. cerebral palsy, spinal cord injury, amputations) (question 8) and a person who is transgender or genderqueer (question 13).

### Summary of attitudes results

This section assessed students’ level of agreement or disagreement to attitudes on 26 sexuality-related opinion statements. It was emphasised that there were no wrong answers. There are 26 unique statements in this section. Table 3 presents the mean and standard deviation of each.

**FIGURE 1:**
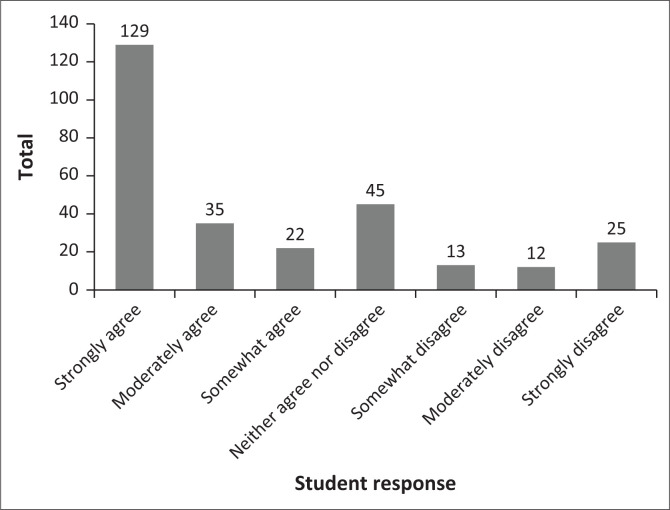
Total attitude score.

**FIGURE 2:**
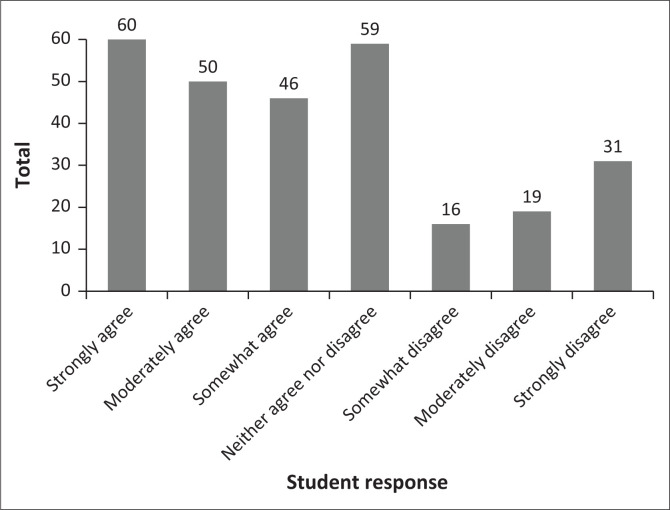
Masturbation is a healthy part of human development.

**FIGURE 3:**
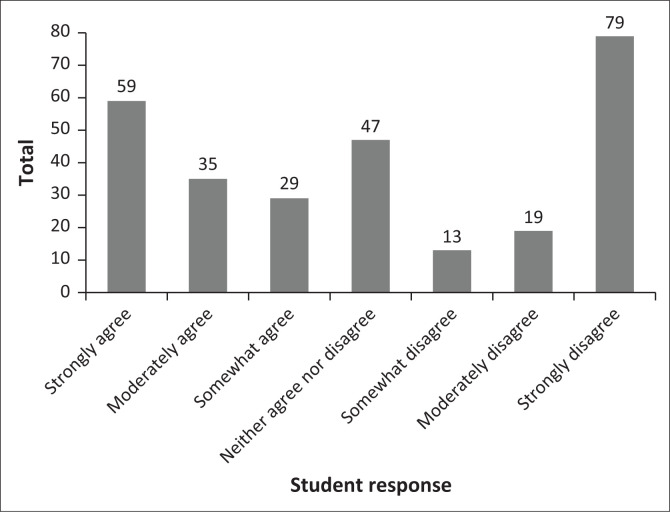
It is okay to have sex before marriage.

The attitudes of the students, described in Table 3, indicate the average opinion of the participants, favouring a perspective of agreement. The data in Figure 1 notes a binomial distribution. Attitudes range from more accepting of sexual behaviour to some students disagreeing. Two examples have been included: (1) ‘Masturbation is a healthy part of human development’ (Figure 2) and (2) ‘It is okay to have sex before marriage’ (Figure 3).

## Discussion

It is the first time that the SHEPS has been used in a South African context and the first time first-year medical students have answered it before they embark on their sexual health course.

The response rate of surveys (when doing research) is usually less than 50%, and during the previous use of the SHEPS questionnaire in a USA study, the response rate was 43%.^21^ Our response rate of 97% might be attributed to the fact that the SHEPS formed a mandatory part of the curriculum, as well as offering a random draw as incentive.

The reason for the uneven gender distribution (Table 1) is not apparent but could be related to the admission policy of the FMHS as well as the global feminisation of medicine.^23,24^ This finding will influence the content development of future modules in the sexual health course, as the answers to the SHEPS questionnaire might differ depending on the different genders in the class, and therefore all genders will be respected in the content development.

**FIGURE 4:**
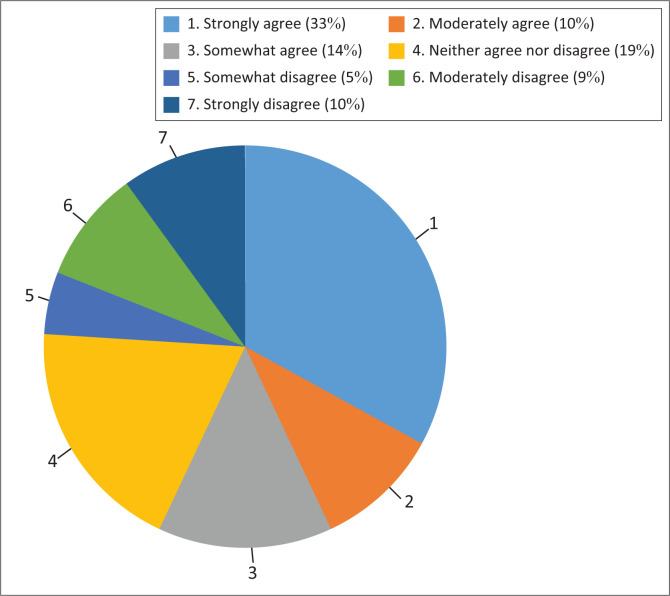
All pornography should be banned: Females.

**FIGURE 5:**
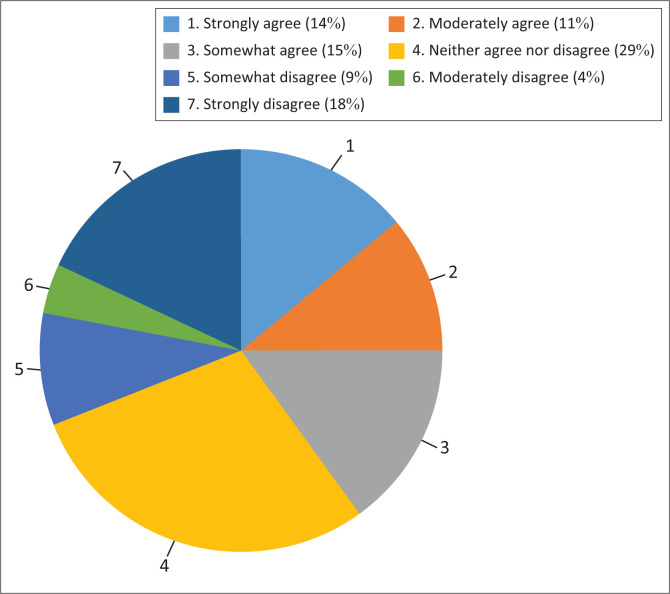
All pornography should be banned: Males.

The gender bias is illustrated by one of the questions in the attitudes section of the SHEPS regarding pornography. The research team noticed the subsequent response distribution: 33% of the female-identified population felt very strongly that all pornography should be banned (Figure 4). In comparison, only 14% of the male participants felt this way (Figure 5). However, findings from previous studies indicate that 1 in 3 females in the 18–30 years age group consume pornography, and this figure has remained consistent from 1973 to 2010.^25,26^ We could expect the gender distribution of this answer to change as the students become older, and move beyond gender conforming attitudes. Nevertheless, understanding the gender dynamics in a classroom will help educators be sensitive with regard to students’ personal and professional development.

As noted in the results section, the correlations between perceived confidence in knowledge and in communication skills were weak to moderate, indicating that comfort in communication and knowledge do not necessarily have a strong relationship at this point in the medical training. Further illustrating this observation, we noticed that the students had more confidence in their communication skills than in the knowledge section, even though they have not yet received formal training in either of these. In Table 1, it is shown that most students (55%) have had some form of sexual health education before medical school, mainly in the 13–18-year age group. The students’ perceived level of confidence in communication and knowledge illustrated their prior learning experience as opposed to course material exposure. Prior learning experience could be because of CSE in schools, the influence of social media and communicating about sex or having discussions with friends, family, schoolteachers and healthcare workers.

These first-year medical students reported a higher than anticipated confidence level in the communication and knowledge questions. This finding can be attributed to the stringent admission criteria to medical school requiring well developed articulation skills. In addition, the average age of the students was 18.7 years, indicating they were admitted to medical school directly from high school. Therefore, we expected them to not have had experience in communicating with a patient in the role of health care provider, and we expected them to score low on perceived knowledge and perhaps communication skills, which include comfort with an area. During their school years, life orientation skills on sexual health are taught for their own development, not to communicate with or assess another person.

What we can take from this discussion and further implement in our sexual health course is that students have a perceived comfort level with regard to sexual health prior to starting medical school. The comfort level of the students was higher than we anticipated, even though they did not have the necessary knowledge yet to support their confidence in communication. Therefore, making sure that we give them the necessary support in building their knowledge base with regard to sexual health will further increase their comfort when having to take a sexual history from their patients.

The attitudes of the students, as noted in the results section and described in Table 3 and Figure 1, favour a perspective of agreement to the statements offered although there are attitudes that range from some more accepting to some students disagreeing (Figure 2 and Figure 3). The attitude distribution of the class provides the insight that the content of each module of the sexual health course should include different viewpoints, taking all the individual attitudes in the class into account.

Culture plays a fundamental part in the health of an individual, a family and the larger community – especially in Africa. How an individual behaves is strongly influenced by the beliefs and standards of the larger family and community.^27^ This has certain implications for the sexual behaviour of the individual – and in the case of medical students, might influence their attitudes with regard to their patient’s sexual behaviour. Attitudes to sexual behaviour of both patients and doctors will be influenced partly by social factors including culture.

Education is the process by which culture is transmitted between generations. It is also the means whereby people learn to appreciate or form value judgements about cultural activities or products. Education is a process of socialisation by which culture is imparted and develops creativity that can challenge existing cultural norms.^28^

The design of the sexual health course keeps this in mind. During the sexual health course, students will be exposed to different opinions about lifestyle, religion, culture and views on sexuality and sexual health care and to health care professional who act as role models. They will be guided towards respectful and professional conduct in these situations by implementing different educational activities. The repeated measurement of the SHEPS at various instances throughout the 6 years of training will indicate the effectiveness of the sexual health course towards the students becoming more comfortable with and including sexual history taking of their patients. The intention is not to change students’ personal beliefs but to teach them about the required professional attitude as a healthcare provider and to understand more of the possibly different context where their patients will be coming from.^21^

In the communication results section, we mentioned some of the areas the students felt most uncomfortable and uncertain about. It was apparent that the students did not yet have the confidence to communicate about sexual health matters with people in age groups other than their own – such as young children and middle-aged adults, especially adults in the >65 age group. They were also less confident in their ability to communicate about sexuality-related topics with a person with a mental disability, a person who engages in non‐normative sexual practices, a person who engages in transactional sex, a person who engages in sex with a person other than a partner in a dyadic relationship without the other partner’s knowledge or consent, a person who is coerced or abused by their sexual partner, a person seeking an abortion and a person who values that about one or several aspects of sexuality conflict with their own.

The question that the study participants were the least confident about was how to communicate about sexuality-related topics with a person who is coercive or abusive to their sexual partner or partners. Many students may not yet have encountered these situations in their lives or have not been taught the necessary tools and communication skills to discuss these topics. To address the discomfort, especially with the topics that they might not have encountered personally, we will include practical exercises where they can practice with standardised patients whom will have the characteristics, they feel less knowledgeable and less skilled in.

The same will also be done for the areas where the students’ perceived knowledge was lacking, which, for the most part, were similar to the communication section, with a moderately positive Pearson’s correlation coefficient. Specific areas of limited knowledge, which did not correspond to the communication section, included knowledge about a foetus or newborn with a disorder of sex development, a person with a physical disability and a person who is transgender or genderqueer. We will take care to include these topics in the appropriate module of the sexual health course.^29,30^

## Conclusion

These baseline data illustrate the wide range of confidence in knowledge and communication skills obtained by prior learning or the lack thereof, before the start of the sexual health course, including data with regard to prior knowledge, skills and attitude introduced by the CSE in schools. The data illustrate the range of perspectives about sexuality students have, with a degree of uncertainty about several of them rather than firm opinions. These attitudes follow a highly positively skewed distribution with the mode at the liberal pole (Figure 1).

The educational team can use the data obtained by the follow-up SHEPS to guide and develop the sexual health course. The SHEPS will elicit areas where medical students are confident and comfortable or hesitant and uncomfortable in dealing with sexuality issues of their patients as health care professionals. These data provide a valuable guide for designing culture sensitive sexual health curricula that attends to both strengths and weaknesses that students bring to their education.

The baseline attitude and perceived communication and knowledge results before starting their sexual health course may also enable us to measure the overall impact and effectiveness of the learning material at the end of the 6 years. Knowing what abilities and limitations students bring into the course is also important to assess what was developed during the course, rather than pre-existing.

No research has yet been done using SHEPS as a tool to guide sexual health curriculum development, and we hope to add valuable information on how using the baseline results of SHEPS can facilitate culture sensitive education.
